# The role and diagnostic significance of cellular barriers after concussive head trauma

**DOI:** 10.2217/cnc-2017-0019

**Published:** 2018-01-31

**Authors:** Aaron Dadas, Damir Janigro

**Affiliations:** 1 FloTBI, Inc, 4415 Euclid Ave Cleveland, OH 44103, USA; 2 Department of Physiology, Case Western Reserve University, Cleveland, OH 44106, USA

**Keywords:** barrier, biomarker, cerebrovascular, imaging, personalized medicine, point-of-care diagnostics, saliva

## Abstract

The onset of concussive head trauma often triggers an intricate sequence of physical consequences and pathophysiological responses. These sequelae can be acute (i.e., hematoma) or chronic (i.e., autoimmune response, neurodegeneration, etc.), and may follow traumas of any severity. A critical factor for prognostication of postconcussion outcome is the pathophysiological response of cellular barriers, which can be measured by several biomarkers of the acute and chronic postinjury phases. We present herein a review on the postconcussion mechanisms of the blood–brain barrier, as well as the diagnostic/prognostic approaches that utilize differential biomarker expression across this boundary. We discuss the role of the blood–saliva cellular barrier as a regulatory filter for brain-derived biomarkers in blood, and its implications for saliva-based diagnostic assays.

Despite an increasing global concern regarding the impact of concussion and traumatic brain injury (TBI), we have yet to achieve a universally accepted definition, list of categorization criteria or management protocol for these ailments. A mild traumatic brain injury (mTBI) or concussion is defined as a complex pathophysiologic process affecting the brain, induced by traumatic biomechanical forces secondary to direct or indirect forces to the head. mTBI is caused by a jolt to the head or body that disrupts the function of the brain. This disturbance of brain function is typically associated with normal structural neuroimaging findings (i.e., CT scan, MRI, etc.). mTBI results in a constellation of physical, cognitive, emotional and/or sleep-related symptoms that may or may not involve a loss of consciousness [1–3]. The medical literature is often inconsistent in distinguishing severity of trauma, however, the labels ‘closed-head injury’, ‘mild traumatic brain injury’ or ‘cerebral contusion’ are used with little differentiation. Even the Centers for Disease Control and Prevention provides a collective and comprehensive definition for concussion and mTBI that uses the two terms interchangeably. However, a number of classifications of TBI exist [4]. In this manuscript, the authors suggest that an ideal classification scheme should be a system that is as simple as possible, but complex and sensitive enough to reliably discriminate TBI into all subtypes.

Concussions in athletes have recently gained a significant boost in popular awareness, due in part to the fact that this injury is a leading cause of brain damage in sports, particularly in American football. Estimates for the frequency of concussions among football players suggest that up to 40% of participants experience a concussion on an annual basis [5], the majority of these going unreported. Currently, these concussions are confirmed or ruled out based on behavioral correlates (e.g., Immediate Post-Concussion Assessment [ImPACT] and cognitive testing) which are used without the aid of standardized laboratory testing. This not only makes diagnosis of sports-related concussions challenging and highly subjective, it creates a risk for misinterpretation and false reporting [6,7]. Although many athletes are unaware of their injury or do not relate their symptoms to a particular event, some avoid medical evaluation altogether for fear of removal from play. For those athletes who do comply with taking a concussion assessment, it is not uncommon for the athlete to sabotage their baseline test, with the intent of falsely presenting a nonconcussive score during in-game assessment. Furthermore, a latency period ranging from several minutes to hours may occur between the traumatic event and development of symptoms, and detailed neurological testing may be delayed for several days after the event. With these confounding factors in mind, the diagnosis of sports-related concussions remains challenging and subjective.

Over 500,000 military veterans have been diagnosed with post-traumatic stress disorder (PTSD), but evidence of brain injury has been reported in 61% of returning soldiers who have been exposed to blast injuries. As a result, TBI has become the ‘signature’ injury of the OIF/OEF troops. As in the case of sports-related TBI, a clear definition for an on-field triage is essential, and the development of adequate tools to quickly determine the severity of concussion are imperative. It remains to be understood, however, how blast-induced TBI differs from the typical civilian impact TBI. Side-by-side studies on biomarkers of these conditions are lacking, but it appears that, as in impact models, the blood–brain barrier (BBB) plays a significant acute and delayed role in blast-induced TBI [8–10].

Despite the varying interpretations of TBI and concussion, it is well understood that an intricate and patient-specific sequence of pathophysiological responses occur in the body after a traumatic impact to the head. Such mechanisms begin immediately at the time of impact and depending on severity of the injury, site of impact and confounding factors of the patient's health may give rise to a range of long-term responses. These symptoms have conventionally been categorized as either occurring in the ‘acute’ or ‘chronic/delayed’ postinjury phases, and will be discussed with this organization in mind in the following sections.

## The blood–brain barrier

**Figure 1.**
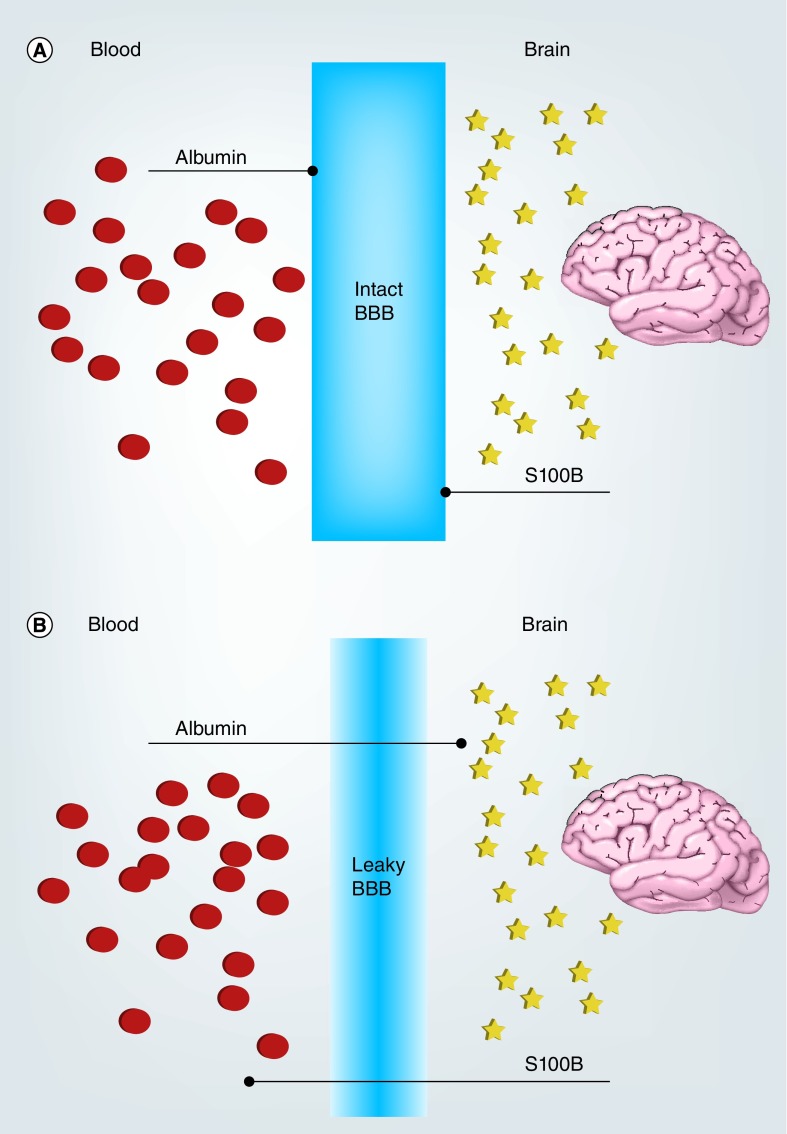
**Serum markers of blood–brain barrier function.** **(A)** In condition of intact BBB, blood-borne and brain-borne proteins cannot extravasate into the other compartment. **(B)** Under conditions of BBB damage, specific brain proteins (e.g., S100B) will extravasate into the blood according to their concentration gradient. Vice versa, serum protein will enter the brain parenchyma (red dots). The quantitative equivalence of serum protein extravasation (albumin quotient) and S100B have been demonstrated. BBB: Blood–brain barrier.

The brain is protected by a main vascular barrier, the BBB. This boundary between the brain parenchyma and systemic blood supply is composed primarily of tight-junctioned endothelial cells (ECs), and maintains variable levels of permeability, or ‘leakiness’, depending on diameter of the vessel [11,12]. The BBB uses selective permeability to protect the brain from harmful substances of the blood stream, while also supplying the brain with nutrients required for proper function and regulating the trafficking of cells and molecules from the blood into the brain. Under homeostatic conditions, the BBB maintains a strict compartmentalization of certain brain-specific proteins from entering systemic blood circulation; if this barrier integrity is compromised (i.e., after a head injury), proteins normally present in high concentrations in the CNS freely diffuse into the blood following their concentration gradients (Figure 1). Understanding these physiological changes in the acute and chronic postinjury phases is a critical factor in the development of diagnostic systems for detecting brain-derived biomarkers in blood.

### Acute mechanisms of blood–brain barrier disruption

A significant postinjury mechanism of concussions, or mTBI in general, is the rapid disruption of BBB integrity; a critical step in its diagnosis is determining whether this disruption is also accompanied by development of brain damage [13]. Thus, there are significant clinical benefits to detecting BBB changes in the acute postinjury phase as an indicator for the patient's propensity toward subsequent brain damage. Identification of this injury-associated barrier disruption has been traditionally conducted with radiologic imaging (e.g., MRI [14]) and laboratory findings such as the albumin quotient, however these methods require patient transport to an emergency room or compliant testing facility. Since physiological changes to the BBB only prevail for a short period of time following concussive head injury, these approaches are perhaps of limited use for a quick determination of the patient's cerebrovascular integrity after mTBI.

An emerging alternative for clinical diagnostics is the testing of serum for brain-derived biomarkers in the acute postinjury phase. Among the candidate biomarkers for such testing is S100B, whose serum levels have been proven to reflect the presence of a damaged BBB, and may predict or rule out brain injury [15,16]. Most importantly, S100B presents a quantifiable increase after an mTBI characterized by computed tomography (CT) changes consistent with intracranial events. In studies where S100B serum levels were compared with CT-based diagnosis of mTBI, a negative predictive value (NPV) of >95% was reported for this biomarker [17,18]. Approaches for on-site detection of blood biomarker levels indicative of brain health are currently being pursued [14,15], however blood-based biomarker testing is met with its own set of limiting factors. Principal among these complications is the need for invasive retrieval of the blood sample, preprocessing/filtration of blood cells and mitigation of nonspecific binding entities. Furthermore, many end user groups such as the National Football League and military have expressed reluctance in incorporating a sideline or field-of-battle diagnostic test that requires blood draw. Thus, the blood-based biomarker approach for acute determination of head injury has yet remained an *in vitro* practice (i.e., Scandinavian guidelines for initial management of head injury), and attention has been geared toward other more accessible fluids for sampling, such as saliva which is later reviewed.

#### The glymphatic system as a source of markers of traumatic brain injury

In addition to the endothelial–brain interface at the BBB, another potential pathway for markers’ extravasation has been recently proposed by Nedergaard's group [19,20]. The authors describe a ‘glymphatic’ system which may remove extracellular protein from the brain parenchyma by an arterial pulsation-driven mechanism. Ths glymphatic system can be summarized as follows:

- The brain is endowed with a waste clearance system driven by bulk flow of fluid through the interstitium;
- The direction of flow is from para-arterial to paravenous spaces;
- The process is facilitated by astrocytic aquaporin-4 (AQP4); animals lacking AQP4 display reduced rate of clearance.

The anatomic localization of this system is still lacking; this, together with the recent discovery of a true lymphatic system in the dura mater of the brain [21] has shed some doubt on the contribution of glymphatic drainage to markers’ extravasation after TBI. Previous studies reported that S100B is increased within minutes after BBB disruption following carotid–jugular endarterectomy [22] or intravenous blood after osmotic disruption [23]. In the study by Nedergaard and colleagues, increased levels were seen only at much later time points showing that the early appearance of markers is not a glymphatic consequence. Also important to note is the fact that passage of S100B in systemic circulation parallels gadolinium extravasation across a leaky BBB [24,25]. At last, blood S100B correlates with albumin cerebrospinal fluid (CSF):blood ratio [26]. Thus, it is likely that TBI markers’ extravasation is trans-BBB rather than glymphatic. There are several reasons for these discrepancies, but it is likely that studies in humans or animals with an intact brain, CSF and choroid plexus lead to different conclusions compared with the highly sophisticated and innovative studies described by Nedergaard's group.

#### Delayed mechanisms of blood–brain barrier disruption

**Figure 2.**
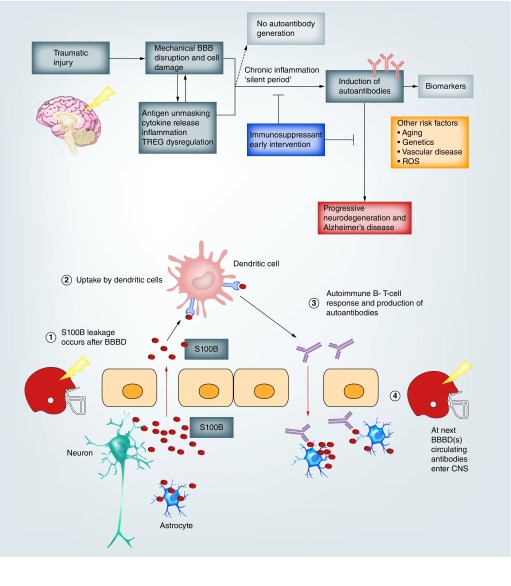
**Putative mechanisms linking repeated head injury to an autoimmune cascade.** Subconcussive, repeated head hits are accompanied by S100B surges leading to production of autoantibodies. Other glioneuronal proteins (e.g., synapsin, various isoforms of MAP associated with tau and neuromodulin) are released in serum after repeated head injuries, which may also lead to an autoimmune response. BBB: Blood–brain barrier; BBBD: Blood–brain barrier disruption; ROS: Reactive oxygen species.

Published data [27–29] collected over several years and from three football teams revealed that persistent structural brain changes occur after repeated head injuries (RHIs). Changes in diffusion tensor imaging (DTI)-MRI and number of RHIs both correlated with levels of serum S100B, as well as the development of an autoimmune response in the form of anti-S100B autoantibodies [27]. We therefore formulated the overarching hypothesis that long-term history of RHIs in athletes may lead to cognitive impairment mediated by an autoimmune response against S100B or yet unknown brain proteins (Figure 2). A recently published paper summarizes the process of S100B uptake in dendritic cells, as well as its distribution in organs of immunity [30].

Immunological self-tolerance is maintained through a diverse range of mechanisms, including active suppression by regulatory cells and deletion of autoreactive immune cells following challenge with autoantigen in the thymus or periphery. A common way to prevent autoimmunity is by hiding self-tissues behind a barrier impermeable to circulating immune cells. Another mechanism is the lack of significant entry of B lymphocytes in normal brain. Indeed, the brain enjoys a conditionally privileged immune status and is normally deficient in local antigen-presenting cells. Brain-reactive T cells, which abound in the healthy immune repertoire but remain innocuous throughout life, can be activated and gain access to their target tissues. The most dramatic consequence of failed immune regulation in the CNS is multiple sclerosis, where immune cells invade the tissue and produce response patterns typical of CNS autoimmune disease. Emerging data indicate that TBI activates B lymphocytes producing antibodies specific for antigen found within and outside the CNS.

The plausibility of this autoimmune hypothesis is supported by multiple prior studies demonstrating a link between barrier disruption, autoantigen leakage, autoantibodies and autoimmune disease. For instance, in sympathetic ophthalmia and Vogt–Koyanagi–Harada syndrome, a delayed autoimmune response to melanin-containing structures in the eye is initiated by leakage of retina-specific protein across the injured blood–retinal barrier causing the loss of function for the damaged and uninjured eye. Further, Kojima *et al*. [31] demonstrated the co-existence of CNS autoantigen and specific autoreactive T cells in the rodent thymus. The autoantigen found in these experiments was S100B. Autoantibodies have been implicated in the pathogenesis of multiple neurologic diseases and the above evidence provides a link between autoimmunity and traumatic insult to a barrier [32].

TBI-induced presence of autoreactive antibodies will impact brain health regardless of how or why the secondary BBB disruption occurs. In other words, presence of anti-CNS IgGs is detrimental in other pathologies, such as stroke [33], dementia [34,35] and epilepsy, etc. [36,37].

## The blood–saliva barrier

### Modeling biomarker kinetics through the blood–saliva barrier

Recently, saliva has become an important biofluid for evaluation of physiological and pathological conditions in human subjects. The use of saliva for diagnostics has many advantages, including simple and noninvasive collection method, little or no need for preprocessing, minimal risk of contracting infections and easy, low-cost storage. An obstacle in the progression of this field, however, has been the lack of a clear-cut understanding for dynamic passage of biomarkers from blood into the saliva. To highlight the prospective advantage of various biomarkers in saliva-based diagnostics, we developed a MATLAB program to simulate the passage of protein from systemic circulation into saliva. This program was originally designed as a physiologically based pharmacokinetic model to describe the distribution of brain-derived biomarkers in blood [13]. Its main structure was expanded to include a new compartment, namely an idealized salivary gland receiving its vascular supply by the external carotid. The venous output was mimicked according to the properties of jugular vein branches. To approximate the combined contribution of protein extravasation along transcellular and paracellular pathways crossing capillary ECs and salivary gland epithelia, we used the following equation to calculate J_S_, the rate of protein transfer from blood into saliva:(1)
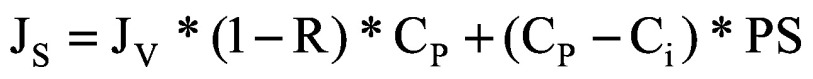



where J_S_ (mol/min) is the mass transfer from blood into saliva; J_V_ (ml/min) represents blood flow to the salivary gland, R is the reflectance of the vascular wall, C_P_ (mol/l) is the concentration of marker in plasma, C_i_ (mol/l) is the concentration of marker in saliva and P and S refer to permeability (cm/s) and surface area of exchange (cm^2^). The value of reflectance has no dimensions, and has a range from 1 (no passage of protein) to 0 (protein passage dictated by diffusion alone). The value of reflectance is derived from pore radius and molecular radius. Most of the values in (1) have been determined experimentally, while the biomarkers’ values in blood (serum or plasma) were derived from our own publications (S100B, UCHL-1) or work by others (GFAP, etc.).

**Figure 3.**
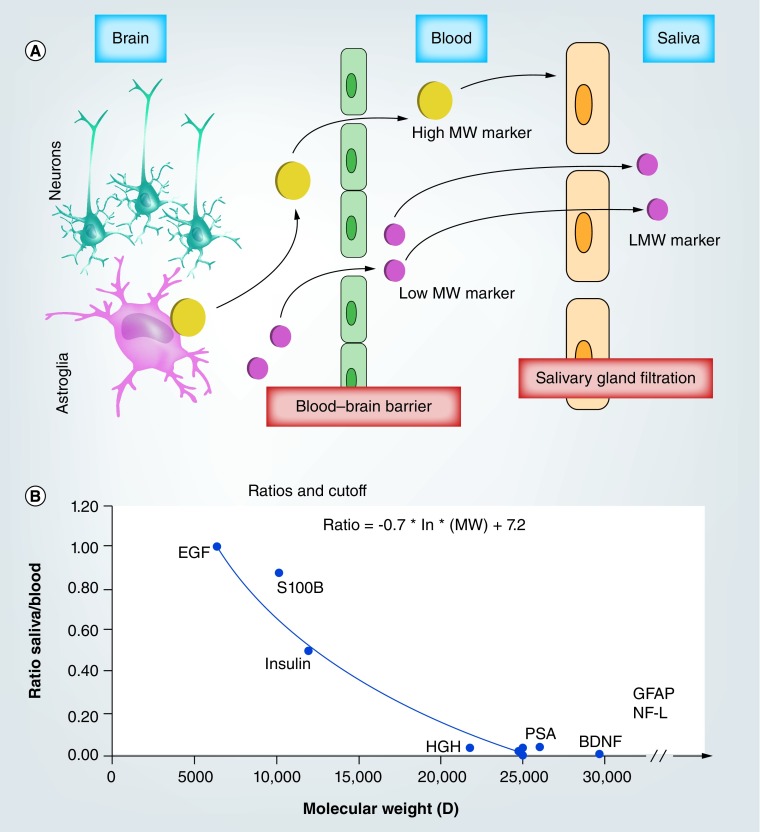
**Simulation of salivary levels of S100B after establishment of blood levels consistent with those measured after traumatic brain injury.** **(A)** Qualitative relationship of protein levels between brain tissue, arterial blood and salivary fluids, demonstrating the selective passage of low molecular weight, brain-derived protein from blood into saliva. See [39,40] for actual validation of this model. See [13] for the description of the model used. **(B)** Summary of salivary cut-off for traumatic brain injury (TBI) markers. Our preliminary results and modeling of biomarker distribution have shown that out of all proteins known to cross the damaged BBB after a TBI, only those with a molecular weight <25 kDa can be found in saliva. To date, S100B is the only TBI salivary marker expected to have excellent negative predictive value. We used the following references to obtain these values: for insulin [41,42], EGF [43], HGH [44], S100B [40,45–47], PSA [48]. There are no reports, to our knowledge, of saliva content of BDNF, GFAP, NF-L. These were plotted to emphasize the cut-off properties of salivary filtration. MW: Molecular weight.

To estimate PS, we used: PS = (J_V_ * C_i_)/(C_P_ – C_i_) with blood flow to salivary gland at 1 ml/min, and C_i_ and C_P_ at 2.5 and 61.5 mg/ml, respectively. These values were derived by measurements of albumin levels in blood and saliva. Recent work [38] demonstrated that the whole saliva proteome displays a larger proportion (14.5%) of low-molecular-weight proteins (< 20 kDa) compared with the plasma proteome (7%). The highest fraction of proteins found in whole saliva and blood range in size between 20 and 40 kDa (26%), whereas the 40–60-kDa range is the largest fraction for plasma (18%). These findings are in agreement with selective permeability between blood and saliva for small-molecular-weight proteins, many of which are candidates for utilization in diagnostic assays (e.g., S100B, GFAP, etc.). Our modeling-based analysis of biomarker passage into saliva further reinforced the concept of selective permeability: these data, seen in Figure 3, show an inverse relationship between protein molecular weight and ratio of protein levels from blood to saliva.

### Variability in salivary protein content

Being able to determine and account for variability in salivary protein content is a prerequisite for the development of saliva as a robust diagnostic and prognostic tool. In this context, it is important that technical variability induced by fluid collection, processing, etc. is kept at a minimum to reproducibly assess sample variability in states of health and disease. Two broad protein families are classified in saliva based on their origin: the majority of salivary proteins are secreted by salivary glands, while a comparably low percentage of proteins derive from capillary leakage, thus directly from the blood stream. These are typically small-molecular-weight proteins which have been shown to appear in saliva at concentrations inversely related to their molecular weight (Figure 3C). The variability of salivary content is due to several nervous and mechanical stimuli, which are clinically defined as unstimulated and stimulated salivary flow. The difference between the two is significant in regard to a finite number of electrolytes (Table 1), but the levels of small-molecular-weight proteins are relatively unchanged. Additionally, the kinetics of blood-to-saliva small-molecular-weight protein exchange remain largely unaltered in the event of traumatic injury. Unlike the blood–brain and blood–CSF boundaries, whose integrity for selective protein passage rely on an uninterrupted lining of tight-junctioned ECs, the blood–saliva barrier does not experience opening in the acute post-TBI phase. One should be cognizant of salivary protein variability due to leakage from open wounds in the mouth that might occur concomitantly with the head trauma being observed.

**Table 1.** **Composition of saliva and serum.**

**Serum**			**Saliva**		

	**Stimulated**	**Unstimulated**	**Difference**		**Difference (%)**
Sodium (mmol/l)	145	5.76	20	-14.24	-247

Potassium (mmol/l)	4	19.47	13	6.47	33

Calcium (mmol/l)	1.4	1.32	1.4	-0.08	-6

Magnesium (mmol/l)	1	0.2	0.15	0.05	25

Chloride (mmol/l)	120	16.40	19	-2.60	-16

Bicarbonate (mmol/l)	27	5.47	16.03	-10.56	-193

Phosphate (mmol/l)	1.50	5.69	2.7	2.99	53

Osmolarity (osm/l)	295.90	54.31	72.28	-17.97	-33

Protein (mg/l)	7000	1630	1400	230.00	14

Water (%)	92	99.55	99.53	0.02	

Data taken from [49] and [50].

## Clinical significance

In today's clinical management of TBI, x-ray CT is the conventional and most frequently used imaging modality for damage assessment diagnosis, especially during the acute phase at early time points after injury. As the name suggests, the technique of CT imaging relies on emission of x-ray beams in operator-controlled thin axial slices along the object subject. The attenuated x-rays are captured and by an x-ray detector positioned on the opposite side that measures x-ray attenuation per voxel through the object. Using this information, a 3D image of the scanned object can be digitally reconstructed. CT is best suited for detection of intracranial bleeds, structural gross tissue damage and abnormal bone fractures (e.g., skull, orbital bones) in the acute setting and is considered to be more sensitive for cases of moderate or severe TBI. CT has shown to be effective in easily detecting macroscopic lesions and physical changes in the skull and brain, including intracranial lesions, cerebral contusions, edema, epidural and subdural hematomas, skull fractures, pneumocephalus and brain swelling [13]. This imaging techniques is thus used to identify those who require immediate neurosurgical intervention and intensive care. However, CT is not highly sensitive in cases of mTBI which is frequently associated with diffuse and microscopic nature of the injury signals. A 2004 report from the WHO mTBI Task Force indicated that CT imaging was able to detect abnormalities indicative of mild subarachnoid hemorrhage, subdural hemorrhage or contusions in only about 10% of mTBI patients, but that 30–80% of mTBI patients would later experience symptoms congruent with post-concussion sequelae [14,15]. The discrepancy between CT abnormalities and eventual symptoms of mTBI indicates that CT does not have sufficient sensitivity for detecting clinically significant damage incurred in mTBI. Additional disadvantages of CT imaging include its availability being limited to hospital settings and the delivery of potentially harmful ionizing radiations (a single scan being equivalent in dosage to 250 chest x-rays). Recent advancements in alternative neuroimaging techniques such as DTI and functional MRI have allowed us to gain further insight into the pathogenesis and prognosis of TBI, by revealing previously undetectable microscopic structural and functional changes in the brain connectome induced by TBI. These changes have been correlated with clinical outcomes and improve the predictive power of outcome models. However, such advanced imaging techniques are currently restricted to research settings, whereas traditional CT and structural MRI techniques are used in clinical situations.

Considering that the radiation dosage from a single CT head scan is equivalent to 250 chest x-rays, it would be advantageous to develop and validate peripheral fluid markers as an alternative diagnostic/prognostic indicator of TBI in cases of acute trauma. The development of extra-axial intracranial hemorrhage is an important consideration following mTBI, and is the primary motivation for performing initial CT imaging studies. Extra-axial hemorrhages (subdural hemorrhage [SDH] and extradural hematoma [EDH]) may change clinical management, as they mandate admission to an intensive care unit and may require neurosurgical intervention. SDH is a not uncommon in mTBI sequelae, and is often associated with rupture of bridging cerebral veins, causing progressive accumulation of blood in the subdural space. Subacute and chronic subdural hematomas are relatively common in patients with mTBI, especially in the geriatric population and in setting of anticoagulation. It is important to accurately diagnose SDH, as an expending mass lesion may necessitate surgery. Moreover, SDH is often associated with breakdown of the BBB and disproportionate tissue inflammation and cerebral edema which may cause progressive neurological deterioration. EDH may also occur after acceleration-deceleration injuries, and is commonly caused by rupture of a meningeal artery. The timely diagnosis of EDH is also of critical importance, as expending hematomas may become life threatening, and progressive neurological deterioration, often after a lucid interval, require neurosurgical intervention. Bleeding into the subarachnoid space after mild TBI may also occur, but is less likely to require surgical intervention. Thus, it is critically important that acute intracranial pathology and bleeding is accurately diagnosed with a noninvasive biomarker-based test.

With respect to serum and CSF markers of severe TBI, extensive literature is available correlating levels of measured biomarkers to trauma severity, cognitive performance and outcome. Among the oldest and most widely studied of these biomarkers is S100B, which has a high NPV for TBI. However, several limitations have complicated the translation of S100B's use into clinical practice. A predominant concern is the fact that S100B has a very poor positive predictive value (PPV). This fact has led to the belief that a multimarker approach would be more powerful than the use of individual brain-derived signals [51,52]. Nonetheless, a very high NPV, even in the setting of a low PPV, is clinically useful for the management of mTBI in particular clinical contexts [17–18,53]. Due to its excellent NPV, and in spite of its poor PPV, serum S100B can inform the decision regarding the need for an emergency department transfer and the necessity of a CT scan in acute postinjury settings. A biomarker with high NPV can lead to reductions in unnecessary, costly emergency department visits and radiation exposure associated with CT. A study of this hypothesis found that low serum levels of S100B (below 0.10 μg/l) had an NPV of 90–100% for a normal CT scan in patients with minor head injuries. This study reinforces the potential of S100B as a biomarker for informing clinical decision-making and in reducing the number of unnecessary CT scans routinely ordered.

Other biomarkers of acute neuronal injury in TBI include neuron-specific enolase (NSE) and the novel brain–derived biomarkers α-II spectrin and UCH-L1. In studies of severe TBI, NSE was found to be elevated in both ventricular CSF and peripheral serum blood, with a higher magnitude of elevation corresponding to higher mortality and a more severe score on the Glasgow Coma Scale regardless of patient's age for both adults and children. However, a major limitation of NSE as a biomarker is its low lack of specificity – largely due to elevation during hemolysis. Despite its name, NSE is also naturally expressed in erythrocytes and may be elevated due to hemolysis or contamination of CSF with peripheral red blood cells. UCH-L1 and α-II spectrin breakdown products are relatively novel biomarkers which are naturally expressed in neurons which have been found to be elevated in CSF and peripheral blood in response to TBI. The elevation of spectrin breakdown products and UCH-L1 (as well as GFAP) have been correlated with the severity of trauma, and it improved the predictive power for the ImPACT outcome calculator in patients with TBI. Fluid biomarkers of acute axonal injury include tau proteins, neurofilament light peptide and phosphorylated neurofilament heavy peptide. In severe TBI, total tau protein levels are elevated in ventricular CSF and levels correspond to lesion size, injury severity and clinical outcomes [14]. However, the high molecular weight of most of these biomarkers make it unlikely that they will be found at measurable levels in saliva.

## Conclusion

TBIs are a clinical challenge due to the complex nature of their acute and delayed pathophysiological mechanisms. Proper identification and management for TBI of all severities require an advanced protocol that involves the tracking of multiple biomarkers indicative of brain health. Although conventional medicine currently turns toward neuroimaging modalities such as CT and MRI for diagnostic procedures, these methods are limited by accessibility, cost and their ability to detect the more subtle – yet undoubtedly significant – sequelae of concussions and mild TBI. A promising direction for clinical diagnosis and prognosis – which avoids many of the hurdles inherent to neuroimaging – is the observation of dynamic changes to cellular barriers in the acute and delayed postinjury phases, with particular consideration for the differential passage of protein biomarkers through these boundaries. Protein biomarkers crossing the BBB into serum, or crossing the blood–saliva barrier into saliva, provide an potentially low-cost and time-efficient method for detection and prognosis of TBI. Many serum biomarkers, such as S100B, UCHL-1, NF-L and GFAP, have proven to be indicative of molecular and biochemical changes induced by TBI [54–57]. Quantitative levels of biomarkers in CSF and serum have also been correlated with injury severity and long-term clinical outcome. The utility of salivary markers is becoming increasingly recognized (see [58] and below). Currently, a need exists for point-of-care devices that complement the detection of these fluid biomarkers of TBI. The refinement of currently identified serum biomarkers, alongside the discovery/validation of salivary biomarkers, could provide a cost effective and reliable method for detection, evaluation and prognosis of TBI, while avoiding many setbacks of the previously discussed neuroimaging procedures.

- TBI is associated with an ‘opening’ of the BBB as measured by the blood–CSF albumin quotient, MRI and surrogate serum markers such as S100B;
- Subconcussive head hits experienced during American football games cause a rapid (minutes to hours) and reversible (within 24 h) increase in serum S100B. These increases are repeatedly seen during subsequent games [27]. Contrast-enhanced MRI confirmed that BBB hyperpermeability is a consequence of head hits in football games [59];
- A statistically significant positive correlation exists between appearance of S100B in the blood and the development of an autoimmune response against the same [27]. In an animal model, we demonstrated that circulating S100B is rapidly taken up by immune dendritic cells, further supporting the hypothesis that brain-derived protein may act as autoantigen [30];
- The presence of autoantibodies in football players correlates with persistent changes in DTI scans, suggesting a link between white matter changes, extravasation of brain protein and an autoimmune response [27,28];
- Subconcussive head hits or TBI lead to a breach of the BBB which exposes both intact CNS proteins, and following cell death, damage-associated molecular patterns to the peripheral immune system in the context of locally enhanced immune activation [27,30];
- An emerging aspect of TBI is an abnormal activation of the systemic immune response. Recently, the occurrence of autoantibodies following TBI and associated BBB disruption has been reported by two independent studies. Interestingly, while one of the studies enrolled patients with TBI, the other focused on subconcussive brain injuries in football players. Both reported an autoimmune response against astrocytic protein suggesting that TBI is sufficient but not necessary to trigger an autoimmune response [27,32];
- An important aspect of these studies is that autoantibodies are a persistent marker of traumatic subconcussive brain trauma since their presence in blood or brain significantly outlasts the antigens that have in the past been used as acute TBI markers (e.g., S100B, GFAP).

**Executive summary**

- Traumatic brain injury and concussion are becoming an increasing concern to global healthcare.
- We are in need of universally accepted classification and management protocols for these ailments.
- Neuroimaging (e.g., computed tomography, MRI, etc.) is currently the most commonly used assessment modality for head trauma, but faces many limiting factors (e.g., cost, availability, radiation exposure, etc.).
- An emerging method for postinjury assessment is the observation of differential expression of brain-derived biomarkers across cellular barriers of the body, which can be observed in blood and saliva.
- The two cellular barriers of focus for this manuscript are the blood–brain and blood–saliva barriers, which show clinically relevant mechanisms of biomarker expression in the acute and chronic post-traumatic brain injury (TBI) phases.
- Detailed understanding of these mechanisms, and implementation of tests for differential biomarker expression in the acute and chronic post-TBI phases, will help to advance our classification and management of TBI while avoiding the many limiting attributes of neuroimaging.
